# Comparing Deep Learning Approaches for Understanding Genotype × Phenotype Interactions in Biomass Sorghum

**DOI:** 10.3389/frai.2022.872858

**Published:** 2022-07-04

**Authors:** Zeyu Zhang, Madison Pope, Nadia Shakoor, Robert Pless, Todd C. Mockler, Abby Stylianou

**Affiliations:** ^1^Department of Computer Science, George Washington University, Washington, DC, United States; ^2^Department of Computer Science, Saint Louis University, Saint Louis, MO, United States; ^3^Donald Danforth Plant Science Center, Mockler Lab, Saint Louis, MO, United States

**Keywords:** deep learning, convolutional neural networks, explainable AI, visualization, single nucleotide polymorphism, phenotyping, sorghum, TERRA-REF

## Abstract

We explore the use of deep convolutional neural networks (CNNs) trained on overhead imagery of biomass sorghum to ascertain the relationship between single nucleotide polymorphisms (SNPs), or groups of related SNPs, and the phenotypes they control. We consider both CNNs trained explicitly on the classification task of predicting whether an image shows a plant with a reference or alternate version of various SNPs as well as CNNs trained to create data-driven features based on learning features so that images from the same plot are more similar than images from different plots, and then using the features this network learns for genetic marker classification. We characterize how efficient both approaches are at predicting the presence or absence of a genetic markers, and visualize what parts of the images are most important for those predictions. We find that the data-driven approaches give somewhat higher prediction performance, but have visualizations that are harder to interpret; and we give suggestions of potential future machine learning research and discuss the possibilities of using this approach to uncover *unknown* genotype × phenotype relationships.

## 1. Introduction

Sorghum is a cereal crop, used worldwide for a variety of purposes including for use as grain and as a source of biomass for bio-energy production. For biofuel production, the goal of both plant growers and breeders is to produce sorghum crops that grow as big as possible, as quickly as possible, with as few resources as possible. Plant breeders produce new lines of sorghum by crossing together candidate lines that have desirable traits, or known genes that correspond to desirable traits.

Understanding the relationship between genetics and traits is key to improving the breeding process, and to understanding of plant biology in general. High throughput phenotyping (Araus and Cairns, 2014) takes advantage of progress in sensor platforms able to measure data about plant growth and traits at large scale to better understand these relationships.

**Figure 1:**
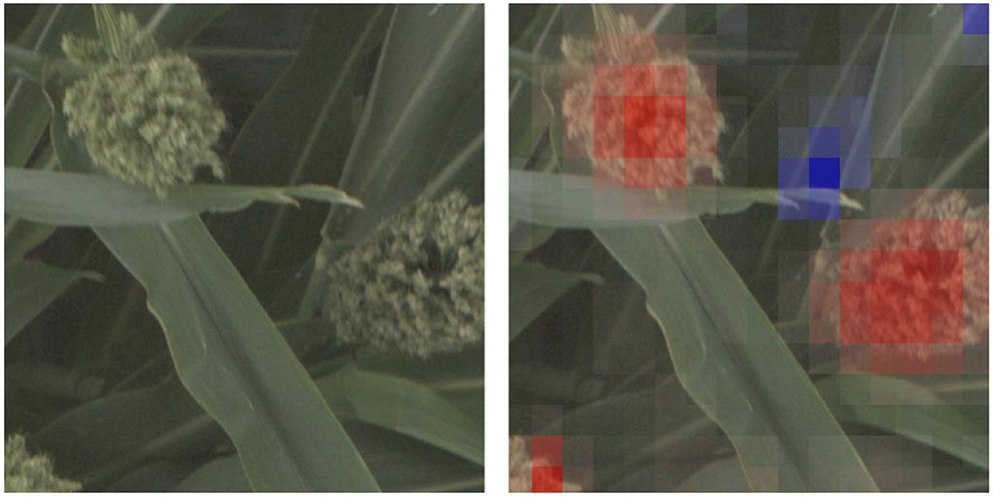
We train deep convolutional neural network classifiers to predict whether an image of a sorghum crop contains a reference or alternate version of particular genetic marker, and then visualize why the network makes that prediction. In this figure, we show the visualization for why the neural network predicted an image showed a plant with an alternate version of a SNP that controls, among other phenotypes, panicle shape (Hilley et al., 2017)—the visualization highlights (in red) the panicle as an important feature in the networks prediction.

In this paper, we propose using deep convolutional neural networks (CNNs) as a computational platform to understand and identify interesting genetic markers that control visually observable traits. The pipelines we present can be leveraged by plant geneticists and breeders to understand the relationship between single nucleotide polymorpishms (SNPs, locations in the organism's DNA that vary between different members of the population), or groups of related SNPs, and the phenotypes that they impact. We explore these genotype × phenotype relationships by training CNNs to predict whether images of biomass sorghum show plants that have reference or alternate versions of different genetic markers, and then making visualizations that highlight the image features that lead to the predictions. For models that can perform this classification task with high accuracy, the visualizations highlight phenotypes that correlate with the genetic marker. Figure 1 shows such a visualization for a genetic marker that controls panicle shape—the visualization shows that the machine learning model learned to focus on the panicles, while not focusing on other plant parts.

We consider two approaches to performing this classification and visualization task. The first approach directly trains a CNN to classify images by their genetic variations. The second approach involves first learning an embedding that can distinguish between different varieties of sorghum, and then training different classifiers on top of that embedding. In both cases, we can quantitatively evaluate how well the models can be used to predict genetic variations and qualitatively assess whether the visualizations provide meaningful and biologically relevant information about the genotype × phenotype relationship.

We demonstrate the feasibility and utility of these pipelines on a number of SNPs identified in the sorghum Bioenergy Association Panel (Brenton et al., 2016) (BAP), a set of 390 sorghum cultivars whose genomes have been fully sequenced and which show promise for bio-energy usage. We focus on SNPs and groups of SNPs with known phenotypic expression in order to validate our approach. We highlight both quantitative results, demonstrating that classification and embedding networks can successfully be trained to predict genetic variation in biomass sorghum, and present example visualizations which highlight that the relevant features learned by these networks correspond to features documented in existing literature about the different genetic markers. The success of this approach on genetic markers with known genotype × phenotype relationships indicates that the same approach could be extended to genetic markers whose phenotypic expression is less well understood, which could help to accelerate crop breeding programs.

## 2. Background

### 2.1. Sorghum and Polymorphisms

Sorghum is a diploid species, meaning that it has two copies of each of its 10 chromosomes. Each chromosome consists of DNA, the genetic instructions for the plant. The DNA itself is made up of individual nucleotides, sequences of which tell the plant precisely which proteins to make. Variations in these sequences, called single nucleotide polymorphisms, can result in changes to the proteins the plant is instructed to make, which in turn can have varying degrees of impact on the structure and performance of the plant. Understanding the impact that specific genes have on plants and how they interact with their environment is a fundamental problem and area of study in plant biology (Bochner, 2003; Schweitzer et al., 2008; Cobb et al., 2013; Boyles et al., 2019; Mural et al., 2021).

Single nucleotide polymorphisms (SNPs) are specific variations that exist between different members of a population at a single location on the chromosome, where one adenine, thymine, cytosine or guanine nucleotide in one plant may be have one or more different nucleotides in a different plant. This variation can exist on one or both copies of the chromosome. A cultivar that has the “original” version of the SNP on both copies of the chromosome is referred to as being homozygous reference; a cultivar that has variant on both copies of the chromosome is referred to as being homozygous alternate; and a cultivar that has one normal and one variant version of the SNP is called heterozygous. In this paper we consider only the homozygous cases, and how deep convolutional neural networks can be used to predict whether imagery of sorghum plants shows a plant with a reference or alternate version of a particular SNP or family of related SNPs.

### 2.2. TERRA-REF

**Figure 2:**
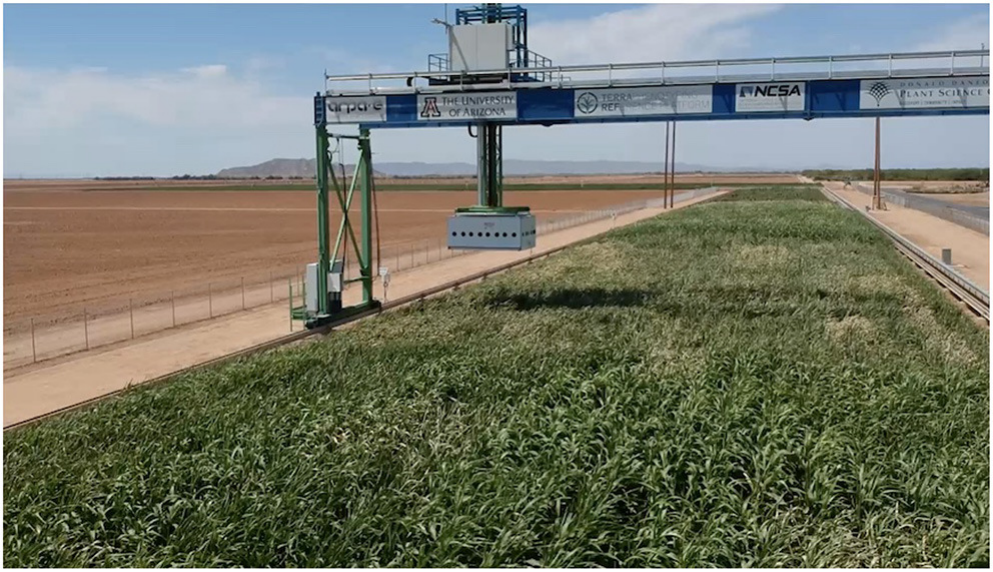
The TERRA-REF Field and Gantry-based Field Scanner in Maricopa, Arizona, with sorghum being grown in the field.

We work with data collected by the Transportation Energy Resources from Renewable Agriculture Phenotyping Reference Platform, or TERRA-REF, project which was funded by the Advanced Research Project Agency–Energy (ARPA-E) in 2016 (Burnette et al., 2018; LeBauer et al., 2020). The TERRA-REF platform is a state-of-the-art gantry based system for monitoring the full growth cycle of over an acre of crops with a cutting-edge suite of imaging sensors, including stereo-RGB, thermal, short- and long-wave hyperspectral cameras, and laser 3D-scanner sensors. The goal of the TERRA-REF gantry was to perform in-field automated high throughput plant phenotyping, the process of making phenotypic measurements of the physical properties of plants at large scale and with high temporal resolution, for the purpose of better understanding the difference between crops and facilitating rapid plant breeding programs. The TERRA-REF field and gantry system are shown in Figure 2.

Since 2016, the TERRA-REF platform has collected petabytes of sensor data capturing the full growing cycle of sorghum plants from the sorghum Bioenergy Association Panel (Brenton et al., 2016), a set of 390 sorghum cultivars whose genomes have been fully sequenced and which show promise for bio-energy usage. The full, original TERRA-REF dataset is a massive public domain agricultural dataset, with high spatial and temporal resolution across numerous sensors and seasons, and includes a variety of environmental data and extracted phenotypes in addition to the sensor data. More information about the dataset and access to it can be found in LeBauer et al. (2020).

### 2.3. Deep Learning for Agriculture

To our knowledge, ours is the first work that trains classifiers on visual sensor data to predict whether an image shows organisms with a reference or alternate version of a genetic marker in order to better understand the genotype × phenotype relationship. There is related work in genomic selection that attempts to predict end-of-season traits like leaf or grain length and crop yield (Sandhu et al., 2021) from genetic information, and in using 3D reconstructions of plants to identify leaf-angle related loci in the sorghum genome (Tross et al., 2021). In Liu et al. (2019), the most related work to ours, the authors train CNNs to predict quantitative traits from SNPs, and use a visualization approach called saliency maps to highlight the *SNPs* that most contributed to predicting a particular trait (as opposed to predicting whether a SNP is reference or alternate, and what visual components led to that classification). There is additionally work that attempts to use deep learning to predict the relative functional importance of specific genetic markers and mutations in plants (Wang et al., 2020), without focusing on visualizing their specific impact on the expressed phenotypes.

There is generally significantly more work in applying deep learning for a wide variety of plant phenotyping and agriculture tasks that do not incorporate the underlying genetics—for example, deep CNNs have successfully been used for fruit detection (Sa et al., 2016; Bargoti and Underwood, 2017; Lim and Chuah, 2018; Koirala et al., 2019; Wan and Goudos, 2020), cultivar and species identification (Barré et al., 2017; Lim and Chuah, 2018; Van Horn et al., 2018; Ashqar et al., 2019; Osako et al., 2020; Heidary-Sharifabad et al., 2021; Ren et al., 2021), plant disease classification (Mohanty et al., 2016; Wang et al., 2017; Ferentinos, 2018; Too et al., 2019), leaf counting (Aich and Stavness, 2017; Dobrescu et al., 2017; Giuffrida et al., 2018; Ubbens et al., 2018; Miao et al., 2021), yield prediction (Wang et al., 2018; Chen et al., 2019; Nevavuori et al., 2019; Maimaitijiang et al., 2020), and stress detection (Anami et al., 2020; Butte et al., 2021; Chandel et al., 2021), among other phenotyping tasks. These deep learning approaches are sensitive to the amount of labeled data available, and the previous works take advantage of a combination of fine-tuning CNN networks trained for other tasks, heroic efforts to hand-label sufficient data to support the learning tasks, or working with existing high-throughput phenotyping data to bootstrap the learning process.

### 2.4. Latent Space Learning and Embedding Networks

When there are too few labels for standard deep learning approaches to work, there are sometimes widely available labels that are still somehow related. These can support alternative ways to train a CNN. One approach is called Deep Metric Learning, and this takes advantages of circumstances when there are sets of images whose labels are unknown, but known to be the same as each other. For example, if you have sets of images that are known to be from the same sorghum cultivar, then you know that those images have the same (but unknown) genetic markers as each other. For such data, deep metric learning trains convolutional neural networks to extract output features from images so that input data from the same class produce similar output features, and input data from different classes produce different output features.

Many approaches to solve this problem have been proposed in recent years, both varying specific loss functions to define the embedding (Hadsell et al., 2006; Sohn, 2016; Ge, 2018; Kim et al., 2018; Xuan et al., 2018), and proposing interesting datasets along with loss functions (Schroff et al., 2015; Song et al., 2016). In this work we use a variation called the Proxy Loss approach described in (Movshovitz-Attias et al. (2017) and Boudiaf et al. (2020), which was recently used for plant-recognition based on flower images (Zhang et al., 2021). This trains an embedding network so that images taken from the same field plot are mapped closer together than images taken from different field plots; this source of weak labeling would apply to any situation where field plots consist of unique cultivars.

The idea of embedding images into a feature space that captures fundamental variations in crop varieties was proposed as “Latent Space Phenotyping” (Ubbens et al., 2020), where the authors used a similar approach to automatically find image features that highlight differentiated response to treatment effects. In their case, the embedding network is trained to learn image features that best capture how the plants in the dataset respond to the experimental treatment (such as drought stress or nitrogen deficiency), to discover image features that might not correlate to standard phenotypes. In our case, we build a network that embeds images into a latent space that helps differentiate many different cultivars, and show that this latent space supports classification of cultivars based on several genetic markers.

### 2.5. Visualization Approaches

A common strategy for making deep convolutional neural networks and their decisions more interpretable is to produce automatically generated visualizations that highlight the most important regions in images for a particular output. There are a variety of different approaches for making these visualizations, including output-agnostic approaches that generate a binary relevancy map by thresholding the values of a feature map from a given layer in the network (Zhou et al., 2015; Bau et al., 2017) or incorporate deconvolutional neural networks to transform activation maps into the original pixel space (Zeiler and Fergus, 2014).

**Figure 3:**
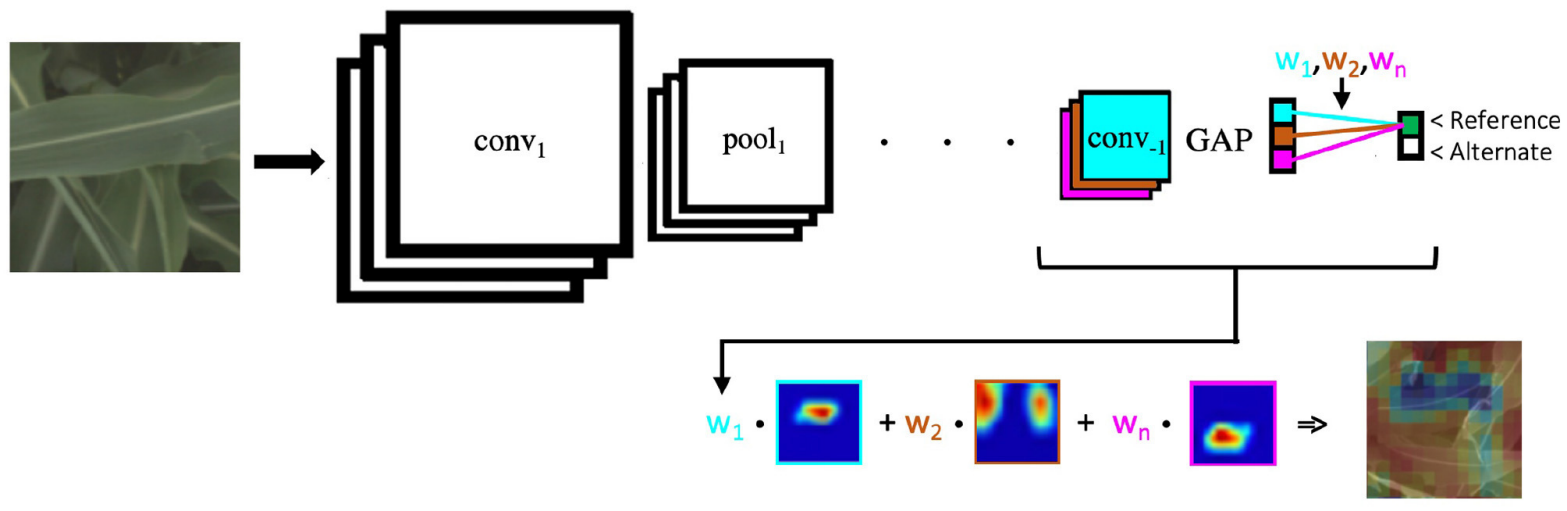
We use a standard ResNet-50 architecture, which like many deep convolutional neural networks consists of alternating convolutional and pooling layers (with interspersed activation functions). The network ends with a final convolutional layer (*conv*_−1_), a global average pooling (GAP) operation, and then a fully connected layer, the output of which is used to make our prediction of whether an image shows a plant with a reference or alternate version of a particular genetic marker. We use the class activation mapping approach described in Zhou et al. (2016), in which the filters in the last convolutional layer are multiplied by the corresponding weights between the respective layer and the predicted output node. These weighted filters are then added up to produce a heatmap that has its highest values in important regions.

One of the most common styles of visualizations that is output-specific is the Class Activation Map (CAM) (Zhou et al., 2016), which were shown to produce discriminative visualizations. CAMs are generated by taking a weighted sum of the feature maps produced by the last convolutional layer in the network, using the weights of the global pooled feature with respect to the target class as a multiplier (as shown in Figure 3. An extension of CAM, GradCAM (Selvaraju et al., 2017) generalizes this framework for different network layers and architectures, weighting the feature maps by the gradients with respect to the target class.

For embedding networks there are fewer visualization approaches. In Chen et al. (2020), the authors extend the GradCAM approach to embedding networks by averaging the gradients from sampled training triplets. To produce the visualization of a test image, the gradients of the most similar training image are used for the weighted sum of the feature maps. In Stylianou et al. (2019), the authors introduce a method for generating heatmaps from a pair of images which highlight the regions that contribute the most to their pairwise similarity by decomposing the similarity calculation across each spatial location in the final feature maps of both images.

In this paper, we focus on the Class Activation Map style visualization to understand the predictions of deep convolutional neural networks relative to particular families of genetic markers in biomass sorghum.

## 3. Dataset Details

To support our study on the usage of deep convolutional neural networks to understand the genotype × phenotype relationship in biomass sorghum, we leverage RGB imagery from the TERRA-REF gantry described in Section 2.2. We specifically focus on images from the 2017 growing season, when cultivars from the sorghum Biomass Association Panel (BAP) (Brenton et al., 2016) were grown. Each cultivar was grown in two spatially separated plots.

The original TERRA-REF dataset provides raw RGB images that are 3296 × 2016 pixels. There are approximately 11 images that mostly or completely image each plot for a given day. In pre-processing the raw imagery for our task, images that cross the plot boundary are cropped into multiple images that each contain pixels of plants from only one plot. This data is then organized into various datasets for our specific task of understanding the genotype × phenotype relationship.

Our study focuses on two different strategies for training CNNs for this task—the first approach directly trains CNNs to classify images as having the “reference” or “alternate” version of a particular genetic marker or family of related SNPs; the second approach first trains a genetic-marker agnostic embedding, where images from the same plot are encouraged to have features that are similar and images from different plots are encouraged to have features that are dissimilar. A genetic-marker specific classifier is then trained on top of the genetic-marker agnostic embedding model. Below we describe the specific datasets used for the classification and embedding tasks.

### 3.1. Classification Dataset

In the classification setting, we train a neural network directly on the task of predicting whether an image fed into the network shows a plant that is homozygous reference or homozygous alternate for a particular genetic markers.

In this paper, we focus on the five genetic markers listed in Table 1. Each genetic marker is defined by one or more related SNPs, which have been identified in prior work as having a particular phenotype that is impacted depending on whether the cultivar being grown has the reference or alternate version of the marker.

**Table 1:** Details about the genetic marker families of interest.

**Genetic marker family**	**SNP details**			
	**Chromosome**	**Gene**	**Position**	**Known controlled phenotype**
Leaf wax	1	001G269200	51,588,525	Wax composition (Uttam et al., 2017)
	1	001G269200	51,588,838	
	1	001G269200	51,589,143	
	1	001G269200	51,589,435	
dw	6	006G067700	42,805,319	Plant height and structure, stem length and internode length (Yamaguchi et al., 2016; Hilley et al., 2017)
	6	006G067700	42,804,037	
Dry Stalk (d) locus	6	006G147400	50,898,459	Plant height and structure, and sugar composition (Xia et al., 2018)
	6	006G147400	50,898,536	
	6	006G147400	50,898,315	
	6	006G147400	50,898,231	
	6	006G147400	50,898,523	
	6	006G147400	50,898,525	
ma	6	006G057866	40,312,463	Flowering time and maturity (Murphy et al., 2014; Wang et al., 2015; Cuevas et al., 2016)
	6	006G004400	2,697,734	
tan	9	009G229800	57,040,680	Pigmentation and tannin production (Wu et al., 2012)

*Single nucleotide polymorphisms are grouped by the phenotypes they control, and classification is performed by genetic marker family. Cultivars are defined as reference if they have the reference version of all SNPs on both copies of the chromosomes, and as alternate if they have the alternate version of all SNPs on both copies of the chromosomes (we do not consider heterozygous cultivars)*.

For a cultivar to be labeled reference for a particular genetic marker, it must have the reference version of all SNPs in the family; cultivars are labeled alternate if they have the alternate version of any of the SNPs in the family—this is because even one polymorphism can significantly impact the phenotype being controlled. (We do not consider heterozygous cultivars.)

For each genetic marker, we then count the total number of reference and the total number of alternate cultivars; the minimum count determines the number of cultivars that are put into the genetic marker family specific training and testing sets— the testing set includes half of the cultivars from whichever class has fewer cultivars, and an equal number cultivars from the more represented class.

We additionally balance our testing set such that there are an equal number of reference and alternate images from an equal number of reference and alternate cultivars (both images and cultivars are randomly selected from the initial test set to guarantee this balance). This guarantees that the performance of a random classifier would be at 50% if predicting either per-image or per-cultivar classification accuracy.

All remaining cultivars are put into the training set, without limiting the number of images per cultivar—this allows us to use a large number of training examples, even if there may be imbalance in the number of images per class (reference vs. alternate) or per cultivar. This imbalance is dealt with at training time by an imbalanced sampler per batch, which selects roughly equal numbers of images from the population of reference and alternate examples.

There is no overlap between the training and testing cultivars.

### 3.2. Embedding Dataset

For the embedding approach, we first train a deep CNN to learn a genetic-marker agnostic representation. To do this, we use all available plot-cropped RGB images from the June 2017 TERRA-REF dataset. These images are labeled by plot. This Embedding Pre-training Dataset contains images from both the classification training and testing set, but no knowledge of the data's genetic marker labels is used to learn the representation.

After the pre-training stage, we are able to then train genetic marker family specific classifiers on top of the embedding model. Details of these classifiers and how they are trained are discussed in more detail in Section 4.2. The test datasets used to evaluate these classifiers are the same as in the classification pipeline. This is acceptable despite the existence of these testing images in the Embedding Pre-Training Dataset as we only use the *plot* labels to pre-train the network; the genetic marker labels are unseen during this stage. Genetic marker dataset splitting that is based on cultivars also assures the plot label pre-training does not force the model to map train and test images together.

Table 2 shows the exact number of cultivars and images used in the classification training and testing sets for each genetic marker family (the Embedding Pre-training Dataset consists of all available plot-cropped images). We only consider images from June of 2017, mid-way through the growing season when plants are not too small, exhibiting many of the phenotypes of interest, and not yet lodging (falling over) on top of each other.

**Table 2:** Dataset statistics.

**Genetic marker family**	**# Train cultivars**		**# Test cultivars**		**# Train images**		**# Test images**	
	**Ref**	**Alt**	**Ref**	**Alt**	**Ref**	**Alt**	**Ref**	**Alt**
Leaf wax	67	114	34	34	6,700	11,400	3,400	3,400
dw	80	105	40	40	8,000	10,500	4,000	4,000
Dry Stalk (d) locus	43	127	21	21	4,300	12,700	2,100	2,100
ma	21	167	10	10	2,100	16,700	1,000	1,000
tan	133	53	27	27	13,300	5,300	2,700	2,700

*The number of cultivars and images used in the training and testing sets for each of the genetic marker families*.

## 4. Methods

Our approach to gaining understanding about the genotype × phenotype relationship in biomass sorghum is to train deep convolutional neural networks to predict whether an image shows a sorghum cultivar with the reference or alternate version of a specific SNP or group of related SNPs, and to then visualize the specific features the network focuses on when making that determination. If the classifier can perform well above chance performance on this classification task, then it is learning something that is significantly correlated with the genetics being considered, and the visualizations can help us glean insights into precisely what those correlations are.

### 4.1. Training Pipeline 1: Classification

We train a ResNet-50 model (He et al., 2016), pre-trained on the ImageNet dataset (Deng et al., 2009), with a single fully connected layer on the reference vs. alternate classification task. A general overview of this type of network architecture is shown at the top of Figure 3.

For all families of genetic markers, the network is trained on 512 × 512 plot-cropped RGB images from the datasets described in Section 3. The weights of the entire network are trained using the adam optimizer (Kingma and Ba, 2015) with a learning rate of 0.0001 for 20 epochs. For data augmentation, we subtract by dataset channel means and divide by dataset channel standard deviations, and during training we perform random horizontal flips. The 512 × 512 pixel images are extracted by resizing the image on its largest side to 512 and extracting a random crop at training time, and a center crop at testing time. We use imbalanced batch sampling during training to fill 100 image batches with a roughly equal number of reference and alternate images per batch, even if there is an imbalance in the number of reference and alternate images in the training set.

### 4.2. Training Pipeline 2: Embedding

#### 4.2.1. Pre-training

As in the classification pipeline, we start from a ResNet-50 model pre-trained on ImageNet. Instead of having a two-dimensional output (as we have in the classification pipeline), the output is 700-dimensional, and the network's task is to correctly classify which of the 700 field plots an image came from.

During the pre-training, we use 25 images per batch, with each image labeled by plot number.

Our embedding network loss function uses a cross-entropy variant of Proxy Loss (Movshovitz-Attias et al., 2017; Boudiaf et al., 2020), optimize the network using SGD (Sutskever et al., 2013) with an initial learning rate of 0.01, learning rate decay of 0.1 every 10 epochs, and a momentum term of 0.9. We train for 40 epochs, stopping based on training loss convergence. We use the same data augmentation strategies as in the classification pipeline.

#### 4.2.2. Genetic Marker Prediction Using Embedding Model

Once this pre-training is complete, we freeze the weights of the network and the plot-level classification layer is chopped off, yielding a network that ends with the 2,048-dimensional output of the ResNet-50's Global Average Pooling (GAP) layer, which we use as our feature embedding. This output of the GAP layer is established to be an excellent representation across datasets and problem domains in Vo and Hays (2019). This embedding feature can then either be used directly in inferring genetic marker labels (for example, using k-Nearest Neighbors) or fed into a classifier (for example, a support vector machine or a new classification head on the pre-trained neural network). We discuss these approaches below.

**k-Nearest Neighbors:** In order to predict a genetic marker label using k-Nearest Neighbors, we first extract the 2,048-dimensional embedding feature for each of the images in both the classification training and the testing sets. For every feature in the test set, we look up its k-nearest neighbors in the training set and infer whether the test image is reference or alternate from the mode of the nearest neighbors. We use the value *k* = 11 in all experiments based on empirical testing.

**Support Vector Machine:** To predict a genetic marker label with a support vector machine, we first extract the 2,048-dimensional embedding feature for each of the images in the classification training and testing sets. We use PCA to reduce the dimensionality of these features from 2,048 to 60, and then use the classification training images and labels to train a support vector machine with a radial basis function kernel, and evaluate performance on the classification test set.

**Classification Head on Embedding Network:** For each genetic marker, we take the pre-trained embedding network and add a fully connected layer with a 2-dimensional output. We fine-tune this fully connected layer using the images and labels from the classification training set (the preceding network weights remain frozen). Performance is evaluated on the classification test set. We use SGD with a learning rate of 0.1 learning rate and 0.1 learning rate decay every 5 epochs during training (with no momentum). We stop training based on training accuracy convergence.

#### 4.2.3. Evaluation Settings

When computing the accuracy of each approach on the classification test set, we can consider accuracy per image, per cultivar and per plot-day. Accuracy per image is computed by simply measuring the average accuracy of predicting the correct label over all images in a test set. Accuracy per cultivar is computed by making per-image predictions for all images from a cultivar in a test set, and selecting the mode from those predictions as the cultivar label. This setting does require knowledge of the test set cultivar labels.

Accuracy per plot-day is computed by taking all of the 2,048-dimensional embedding features from a specific plot on a specific day and averaging them together to produce a plot-day embedding feature. This feature can then be used in place of the original embedding features as the input to the k-Nearest Neighbor or SVM classification (this setting is not applicable for the approach where a fully connected layer is added to the embedding model and trained for each genetic marker).

We discuss the relative classification accuracy of each of the genetic marker prediction approaches and each of the evaluation settings on the genetic marker classification task in Section 5.1.

### 4.3. Visualization Pipeline

It is not our ultimate goal to merely show which of the above strategies yields the highest quantitative performance at predicting whether an image shows a plant that has the reference or alternate version of a particular genetic marker. Instead, we hope to clarify the genotype × phenotype relationship that each of these genetic markers. In order to do this, we propose to automatically highlight the visual features that the neural networks learn are most important in accurately predicting reference vs. alternate. Those visual features are correlated with the genetic markers, and reviewing them can provide insights about what phenotypes the genetic markers are controlling.

In order to make such visualizations, we use the Class Activation Mapping approach described in Zhou et al. (2016), which highlights the image regions that most contributed to a classification of the neural network. This approach is detailed in the bottom of Figure 3, where the filters in the last convolutional layer are multiplied by the corresponding weights between the respective layer and the predicted output node. These weighted filters are then added up to produce a heatmap that has its highest values in important regions (e.g., the red regions in Figure 1). We use this approach to compare the predictions among different methods on a particular genetic marker family to understand the different visual traits correlated with being either reference or alternate.

We are able to use this visualization strategy both for the classification pipeline, as well as the version of the embedding pipeline where we train a genetic marker specific fully connected layer at the end of the embedding network. We compare the visualizations from these different approaches and discuss the biological relevance of them in Section 5.2.

## 5. Results

### 5.1. Genetic Marker Prediction Accuracy

In Table 3 we show the test set classification accuracy for all five genetic markers using both the classification and embedding pipelines. We compute the accuracy per image as well as the accuracy achieved by taking the mode of the predictions from all images of a cultivar, as described in Section 4.2.3. Taking the mode per cultivar outperforms the per image accuracy for all but the ma genetic marker. This is possibly due to the large imbalance in the number of images per class in the ma training set (the ratio between reference and alternate images of ma is 1:8, as seen in Table 2). This significant imbalance may lead the classifiers that utilize the training set (the k-NN and SVM approaches) to be biased toward predicting the alternate class, resulting in roughly chance performance.

**Table 3:** Classification accuracy by image and by cultivar.

**Genetic marker**	**Classification**		**Embedding + k-NN**		**Embedding + SVM**		**Embedding + fc**	
	**Image**	**Cultivar**	**Image**	**Cultivar**	**Image**	**Cultivar**	**Image**	**Cultivar**
Leaf Wax	0.611	0.706	0.641	0.632	0.656	0.647	**0.668**	***0.721***
dw	0.600	0.650	0.660	***0.750***	**0.676**	0.738	0.655	0.713
d locus	0.642	0.762	0.669	0.667	0.665	0.667	**0.734**	***0.833***
ma	0.629	0.600	0.556	0.500	0.570	0.500	**0.630**	***0.650***
tan	0.646	***0.796***	**0.682**	0.741	**0.682**	0.704	0.667	0.704

*For each genetic marker, we compare the accuracy of the direct classification approach with each of the approaches that use the embedding pre-training [k-NN, SVM and adding a fully connected (fc) layer]. Accuracy per image is computed on each image in the test set separately. Accuracy per cultivar is computed by taking the mode of the image predictions from each cultivar. The test set for each genetic marker family is balanced such that the classification accuracy by both image and by cultivar are 0.5. For each genetic marker, the highest accuracy per image is shown in bold text, while the highest accuracy per cultivar is shown in italicized bold text*.

Overall the best classification performance is achieved by the approach where we train a fully connected layer on top of the pre-trained embedding model for each genetic marker. This indicates, for single genetic marker prediction task, the embedding network extracts richer features than the direct classification approach.

#### 5.1.1. Per Plot-Day Results

As discussed in Section 3, there are multiple images per plot on any given day in the dataset due to the configuration of the TERRA-REF field and imaging protocols. Any one of these pictures shows only a subset of the plants in a specific plot, and it may be the case that one picture contains relevant visual features for the plot that are not present in a different picture (e.g., one picture might show a particularly indicative panicle while others do not). This suggests that an approach that aggregates features across all of the images from a plot could achieve superior performance.

In Table 4, we compare the accuracy of the SVM approach using the embedding features for individual images as input vs. using plot-day aggregated features (generated using the average pooling described in Section 4.2.3) as inputs in both training and testing. This plot-day aggregation over all of the images from a plot yields significant improvement for all of the genetic markers. The most noticeable improvement comes from the ma marker. This indicates that the most important visual features for the ma marker may only be present in a subset of the plot images.

**Table 4:** Comparison with per plot-day features.

**Genetic marker**	**Per image**	**Per plot-day**
Leaf wax	0.656	0.699
dw	0.676	0.685
Dry Stalk (d) locus	0.665	0.741
ma	0.570	0.761
tan	0.682	0.733

*Comparison of the accuracy of the SVM classification approach using the embedding features for individual images as input vs. using plot-day aggregated features (generated using the average pooling described in Section 4.2.3) as inputs*.

This significant improvement in classification accuracy for the SVM approach, suggests that it would be beneficial to similarly aggregate features across all of the plot images in the pipeline where we train a fully connected layer on top of the pre-trained embedding. While we cannot use the same average pooling of the embedding features that we employ in this paper, one possible approach for such cross-image aggregation was described in Ren et al. (2021), and presents an interesting direction for future work.

### 5.2. Visualizations of Genetic Markers

In the following sections, we discuss the visualizations produced by the classification models. We focus on the biological relevance of the produced visualizations, as well as a comparison between the visualizations produced by the direct classification model vs. the embedding model.

#### 5.2.1. Visualizations From Classification Network

**Figure 4:**
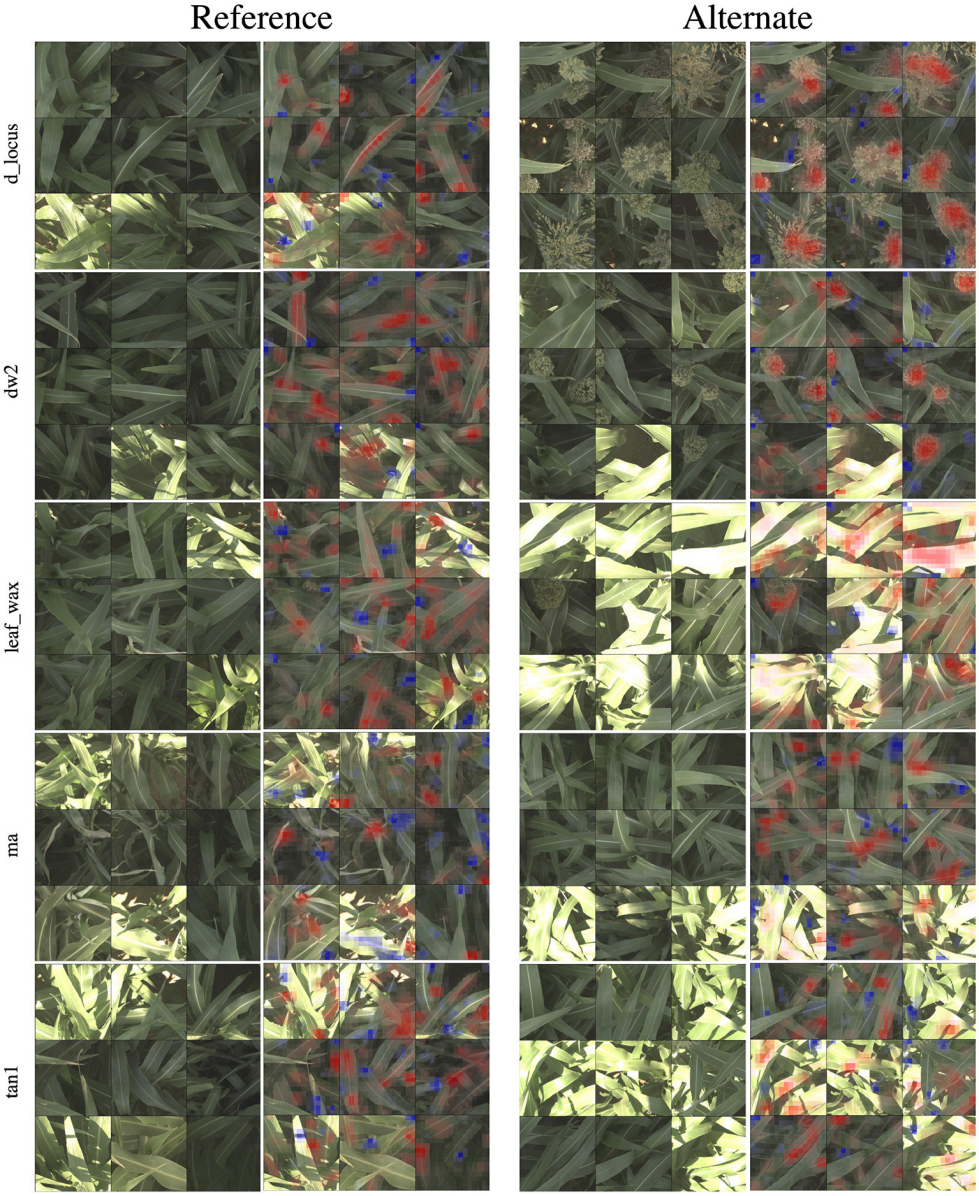
For all five families of genetic markers, we can visualize highly activated and correctly classified images from the “reference” and “alternate classes,” and their corresponding classification visualization that highlights the features that led to the networks classification. Features highlighted in red are those that led the network to make its correct classification, while features in blue are those which detracted from the correct classification.

In Figure 4, we show 9 of the most activated and correctly predicted reference and alternate images and their corresponding heatmaps for each of the genetic markers (limiting our selection to images that aren't extremely over-saturated or under-exposed). These visualizations provide compelling insights into what the networks have learned to focus on, and therefore what visual plant features are highly correlated with a plant either being reference or alternate for a particular genetic marker. In the following paragraphs, we will discuss notable observations from these visualizations and how they correspond to the phenotypes these markers are known to control. In all visualizations, red regions indicate visual features that are important in leading to the correct classification, while blue regions actively detract from the correct class.

In the d_locus and dw visualizations in Figure 4, the alternate visualizations appear to frequently focus on particular panicles at different growth stages (the panicles focused on for the dw and ma genetic markers are earlier in their life cycle when compared to the panicles in the d locus visualizations). This corresponds to the knowledge that polymorphisms in these genetic markers control features like plant growth rate (SNPs in the dw and d_locus families are considered “dwarfing” markers, controlling growth rate and ultimate plant height), flowering time and maturity. The d_locus reference visualizations also appear to focus on particular leaf shapes—the ends of broad leaves—which similarly may relate to the fact that the markers are known to exhibit control over plant structure, and the mid-rib of the leaf. This is consistent with existing knowledge about the phenotype controlled by the d_locus marker as described in Xia et al. (2018): “Dry Stalk (D) locus controls a qualitative difference between juicy green (dd) and dry white (D-) stalks and midribs, and co-localizes with a quantitative trait locus for sugar yield.”

In the leaf wax visualizations in Figure 4, we see the most confident correct predictions for the leaf wax genetic marker family. Cultivars with the reference version of these SNPs are known to be more waxy, while the alternate versions are less waxy. In the reference heat maps, the important (red) regions are often diffuse, covering much of the leaf, while the alternate visualizations are very focused on the spine of the leaf.

**Figure 5:**
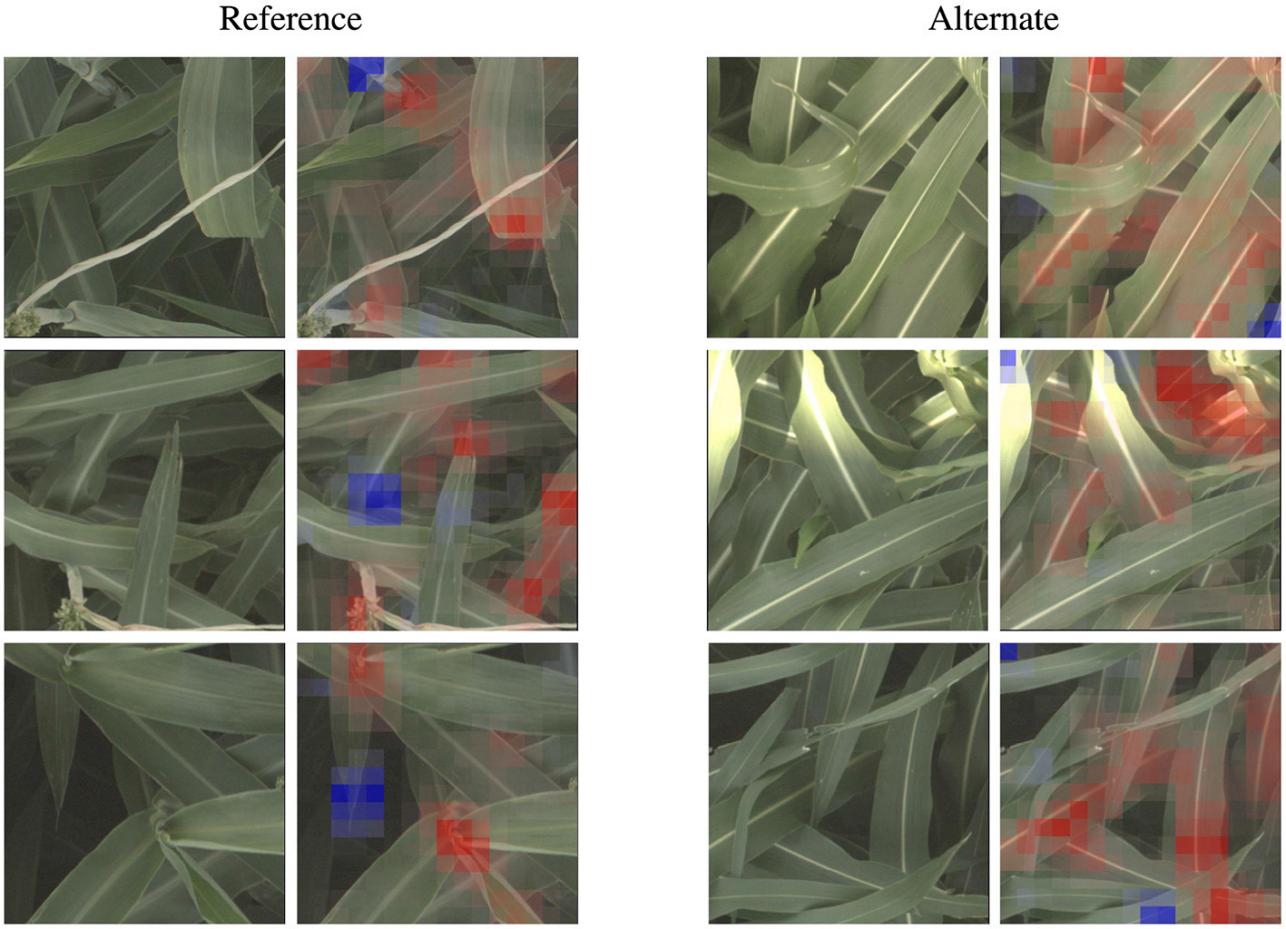
The classification network trained on the leaf wax SNPs learned to focus on specific features for the reference and alternate class. When classifying an image as the higher wax content “reference” class, the network often focuses on the interface between the stem and either leaves or panicles, where the wax build up is most high. When classifying an image as “alternate”, the network instead often focuses on the vivid mid-vein of the leaf that is more obvious when leaf wax content is lower. These features correspond to phenotypes that field biologists observe in the field. Features highlighted in red are those that led the network to make its correct classification, while features in blue are those which detracted from the correct classification.

We zoom in on a selection of these leaf wax images in Figure 5, where it is apparent that in the alternate images, this spine is more brightly differentiated from the rest of the leaf, while in the reference images the spine has less contrast. This corresponds to the wax build up on the leaf in the reference images, which cause the overall leaf to be whiter, resulting in lower contrast on the spine. The reference visualizations also often focus specifically on the interface between the sorghum plant spine and leaf. When reviewing these visualizations with a biologist on our team that does in-field ground truth phenotyping of traits including leaf wax, they said: “That's exactly the place I look at when determining waxiness in the field—it's where the wax is most obvious!” Excitingly, this indicates that the network has learned, without explicit direction, to focus on the same plant parts as expert humans.

In the ma visualizations in Figure 4, we see reference heat maps that highlight the ends and edges of leaves that are old, damaged or browning, and the alternate heatmaps show red highlights on the edges of smoother, apparently healthier leaves, which correlates with impact of this particular genetic marker on the growth stage and maturity of the plants, or the “time to maturity” described in Wang et al. (2015) to be controlled in part by the ma genetic markers.

#### 5.2.2. Visualizations From Embedding Networks

**Figure 6:**
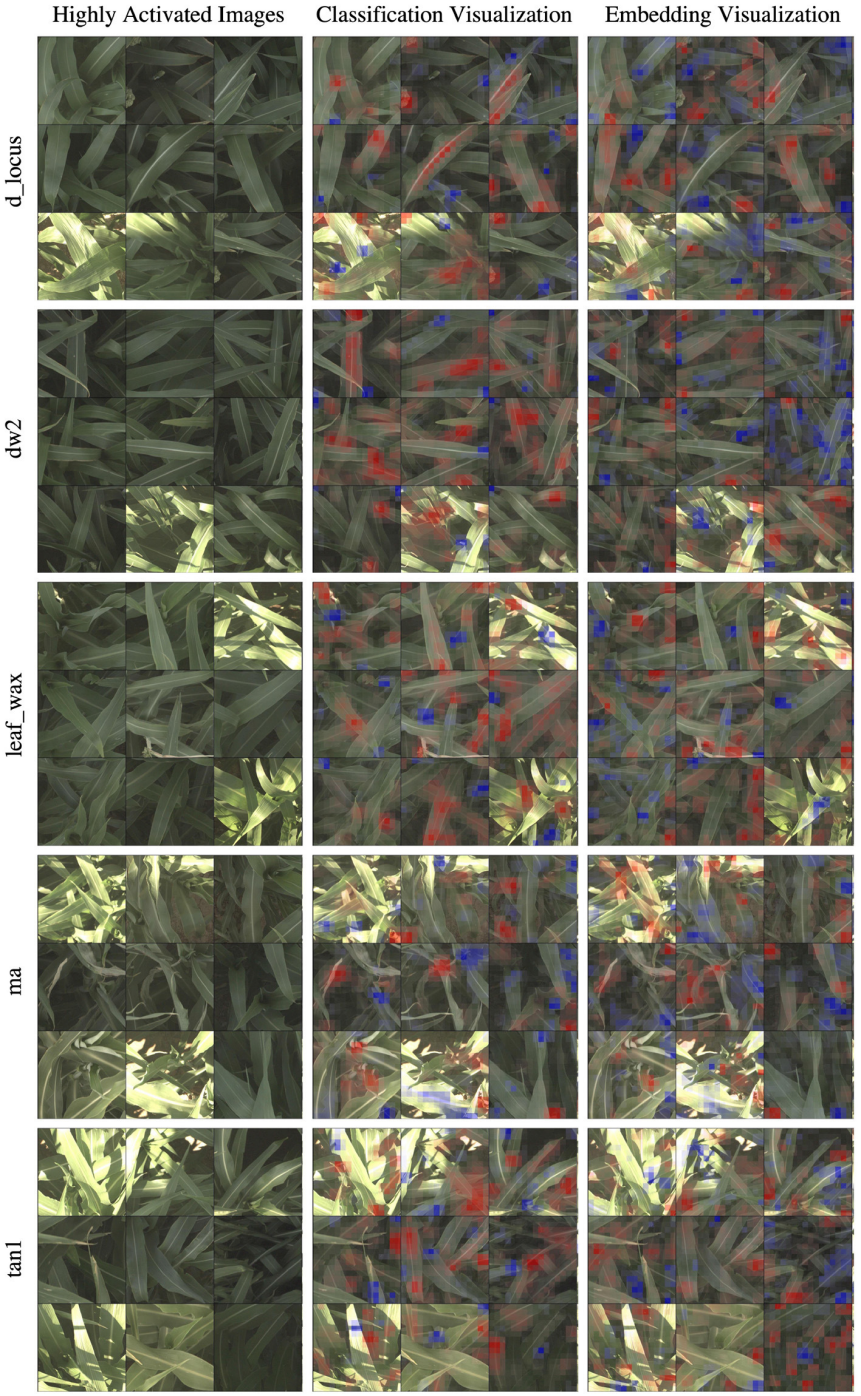
In this figure, we compare the “reference” visualizations from the classification and embedding models over all of the markers. Features highlighted in red are those that led the network to make its correct classification, while features in blue are those which detracted from the correct classification. In general, the classification visualizations focus on specific and more readily identifiable features, while the embedding visualization appears to encompass more diverse but less obvious features. Specific examples of this for the d_locus marker are highlighted in Figure 7.

In Figure 6, we show the same nine highly activated reference images from Figure 4, however this time we show both the visualization produced by the classification model and the visualization produced by the embedding model. While the embedding-based approach achieves higher accuracy, as discussed in Section 5.1, the visualizations are generally less coherent. The classification visualizations often focus on specific and isolated visual features, such as a single panicle or the vein down the center of a leaf.

By comparison, the contributions to the correct prediction highlighted by the embedding visualizations are often much more scattered, highlighting various different visual features simultaneously. The embedding features are trained for the more difficult task of differentiating images of plants in different plots that may look overall quite similar. It is likely that the features learned by the network are good in the aggregate, but individual features may represent combinations of image properties (e.g., “bright midline or wavy leaves or dark shadows”) that are more broadly active across the image. The stronger classification results of the embedding features suggests that it is learning more comprehensive visual features; but additional work may be necessary for this improved performance to also include more interpretable visualizations.

**Figure 7:**
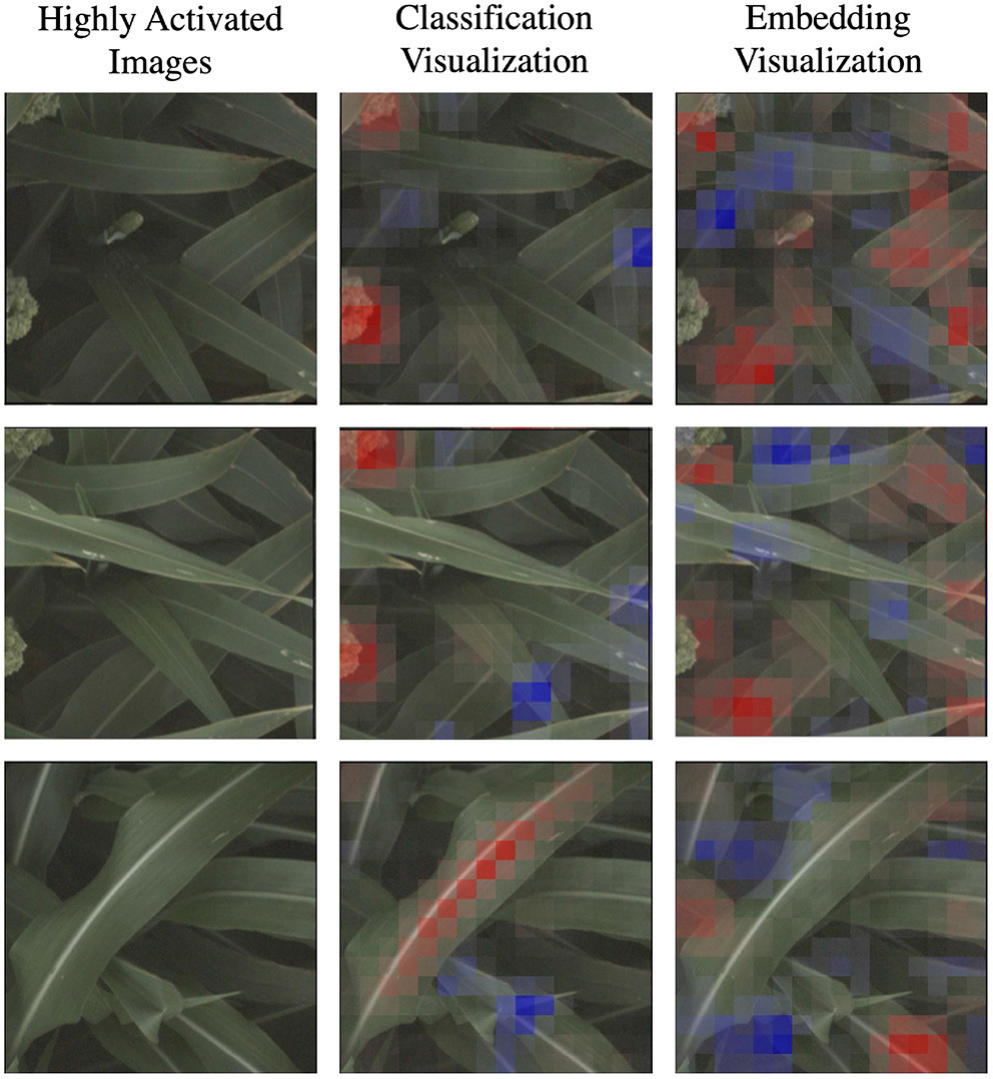
Here we focus on a comparison of the classification and embedding visualizations for highly activated reference images for the d_locus genetic marker family. Features highlighted in red are those that led the network to make its correct classification, while features in blue are those which detracted from the correct classification. This classification visualization clearly highlights specific features such as the panicle or the vein down the center of the leaf, while the embedding visualizations are more diffuse, indicating that the model achieves its higher accuracy by learning more varied (but less readily interpretable) features.

In Figure 7, we highlight three specific examples for the d_locus marker (reference class) that show this difference in the coherence of the visualizations. The classification visualization clearly focuses on panicles in the first two examples and on the leaf mid-rib in the third; by comparison, the embedding visualization on the other hand highlights various parts of multiple leaves in all three examples. In addition to the classification visualization showing consistent, specific features like the mid-rib and panicles, it highlights a relatively small amount of the image as affecting the classification (either positively or negatively). In contrast, the embedding visualizations shows more overall regions of the image with small amount of impact on the classification.

## 6. Conclusions and Future Work

In this paper, we compare two different pipelines to understand the genotype × phenotype relationship in sorghum. The first pipeline directly creates an image classifier by training on images of cultivars with and without a particular genetic marker, and the second trains an embedding that differentiates a wide variety of cultivars and then uses features in that embedding to predict the presence or absence of genetic markers in images of specific plants. We show the embedding approach has an overall better accuracy on genetic marker prediction tasks.

We also visualize the network by showing activation maps which highlight the most important parts of the images that led to the decision of the network. For several genetic markers, the classification approach leads to maps that seem to give clear explanations, as shown, for example, in Figure 5. However, the activation maps created in the embedding approach are more complicated. This is because the embedding network learns features to differentiate many different plots instead of features focused entirely on differentiating one genetic marker. Because each feature may contribute to differentiating many different plots, it may represent a mixture of different kinds of image features and therefore be less interpretable. In future work, a finer grain visualization tool like the one proposed by Zhao et al. (2021) may help to understand and explain the visual features that extracted by the embedding network, and loss functions that encourage sparse representation may make those features more interpretable. Additionally, it may be beneficial to consider visualization strategies that do not simply localize the most salient features, but rather try to disentangle their semantic relevance, such as in the Explaining-in-Style approach proposed in Lang et al. (2021).

We demonstrated the feasibility of our pipeline to help understand the genotype × phenotype relationship in sorghum by training deep convolutional neural networks on visual sensor data to predict whether different crops have reference or alternate versions of particular genetic markers. We show for several genetic markers that whose phenotypic expression is well understood that these networks can achieve well-above chance performance on this task, and that visualizations that highlight the most important parts of the images that led to the classification correspond with the known phenotypes.

This approach can be extended to not only help better understand well-established genotype × phenotype relationships, but to explore new, less well understood relationships. The same approach could be deployed for SNPs and families of SNPs whose phenotypic expression is *not* understood, to uncover the importance of new, unstudied polymorphisms. Such discovery would be achieved by first starting with a list of candidate SNPs from sequencing whose phenotypic expression are not well understood; then, for each one, a classifier would be trained to predict whether images show a plant with the reference or alternate version. If a classifier achieves significantly above random-chance performance on this task, then there is some visual feature that is correlated with the marker. The visualizations of the most salient features for the classifier can then be used to determine precisely what the most important plant features are for that genetic marker, to help drive understanding of these as yet unknown genotype × phenotype relationships. We acknowledge that this approach is limited in terms of determining causation as opposed to correlation—there are often substantial correlations between genetic variation in cultivars making it challenging to attribute changes to individual mutations. However, even correlations provide useful evidence for an investigator seeking to better understand the genotype × phenotype relationship. The pre-trained embedding models that achieved high performance in this study could be used in these explorations of new genotype × phenotype relationships, and our pre-trained models and training code are available in our GitHub code repository, which can be found at https://github.com/GWUvision/sorghum-snp-classification. If an investigator is seeking to generalize this pipeline to new species or to sorghum lines and phenotypes that are not present in the BAP, it may be necessary to re-train on representative data.

In this paper, we focused on a relatively limited time period of high resolution data from the TERRA-REF gantry system (data from the entire month of June, mid-way through the growing season in 2017). We recognize that not all phenotypes, however, are observable during this time period. Especially when considering unknown genetic markers, it may be beneficial to consider longer time periods including both early and late growing periods when different phenotypes are expressed. This is a direction for future work: longer time periods may require more complex training protocols that more explicitly incorporate time—for example, using recurrent approaches, or training a multi-headed network that simultaneously predicts the genetic class and the date. Additional work could focus on extending the approach to sensors other than RGB cameras, as some phenotypes may be more readily observed in different sensing modalities, such as hyperspectral or thermal imagery, or in the structural information from the 3D laser scanner.

## Data Availability Statement

Publicly available datasets were analyzed in this study. This data can be found here: https://github.com/GWUvision/sorghum-snp-classification.

## Author Contributions

AS, NS, RP, and TM contributed to conception and design of the study. AS and ZZ performed analyses. MP performed literature review. NS and TM provided review of biological relevance of visualizations. AS, RP, and ZZ wrote sections of the manuscript. All authors contributed to manuscript revision, read, and approved the submitted version.

## Funding

This work was supported by the Advanced Research Projects Agency-Energy (ARPA-E)/US Department of Energy under grant numbers DE-AR0000594 and DE-AR0001101.

## Conflict of Interest

The authors declare that the research was conducted in the absence of any commercial or financial relationships that could be construed as a potential conflict of interest.

## Publisher's Note

All claims expressed in this article are solely those of the authors and do not necessarily represent those of their affiliated organizations, or those of the publisher, the editors and the reviewers. Any product that may be evaluated in this article, or claim that may be made by its manufacturer, is not guaranteed or endorsed by the publisher.

## References

[B1] Aich S. Stavness I. (2017). “Leaf counting with deep convolutional and deconvolutional networks,” in IEEE/CVF Conference on Computer Vision and Pattern Recognition Workshops (CVPRW) (Honolulu, HI: IEEE), 2080–2089.

[B2] Anami B. S. Malvade N. N. Palaiah S. (2020). Deep learning approach for recognition and classification of yield affecting paddy crop stresses using field images. Artif. Intell. Agric. 4, 12–20. 10.1016/j.aiia.2020.03.001

[B3] Araus J. L. Cairns J. E. (2014). Field high-throughput phenotyping: the new crop breeding frontier. Trends Plant Sci. 19, 52–61. 10.1016/j.tplants.2013.09.008 24139902

[B4] Ashqar B. A. Abu-Nasser B. S. Abu-Naser S. S. (2019). “Plant seedlings classification using deep learning,” in International Journal of Academic Information Systems Research (IJAISR) (Bowling Green, KY).

[B5] Bargoti S. Underwood J. (2017). “Deep fruit detection in orchards,” in IEEE International Conference on Robotics and Automation (ICRA) (Singapore: IEEE), 3626–3633.

[B6] Barré P. Stöver B. C. Müller K. F. Steinhage V. (2017). Leafnet: a computer vision system for automatic plant species identification. Ecol. Inform. 40, 50–56. 10.1016/j.ecoinf.2017.05.005

[B7] Bau D. Zhou B. Khosla A. Oliva A. Torralba A. (2017). “Network dissection: quantifying interpretability of deep visual representations,” in Proceedings of the Conference on Computer Vision and Pattern Recognition (Honolulu, HI: IEEE), 6541–6549.

[B8] Bochner B. R. (2003). New technologies to assess genotype-phenotype relationships. Nat. Rev. Genet. 4, 309–314. 10.1038/nrg1046 12671661

[B9] Boudiaf M. Rony J. Ziko I. M. Granger E. Pedersoli M. Piantanida P.. (2020). “A unifying mutual information view of metric learning: cross-entropy vs. pairwise losses,” in Proceedings of the European Conference on Computer Vision, 548–564.

[B10] Boyles R. E. Brenton Z. W. Kresovich S. (2019). Genetic and genomic resources of sorghum to connect genotype with phenotype in contrasting environments. Plant J. 97, 19–39. 10.1111/tpj.14113 30260043

[B11] Brenton Z. W. Cooper E. A. Myers M. T. Boyles R. E. Shakoor N. Zielinski K. J.. (2016). A genomic resource for the development, improvement, and exploitation of sorghum for bioenergy. Genetics 204, 21–33. 10.1534/genetics.115.183947 27356613 PMC5012387

[B12] Burnette M. Kooper R. Maloney J. D. Rohde G. S. Terstriep J. A. Willis C.. (2018). “TERRA-REF data processing infrastructure,” in Proceedings of the Practice and Experience on Advanced Research Computing, ed S. Sanielevici (New York, NY: ACM).

[B13] Butte S. Vakanski A. Duellman K. Wang H. Mirkouei A. (2021). Potato crop stress identification in aerial images using deep learning-based object detection. arXiv preprint arXiv:2106.07770. 10.1002/agj2.20841

[B14] Chandel N. S. Chakraborty S. K. Rajwade Y. A. Dubey K. Tiwari M. K. Jat D. (2021). Identifying crop water stress using deep learning models. Neural Comput. Appl. 33, 5353–5367. 10.1007/s00521-020-05325-4

[B15] Chen L. Chen J. Hajimirsadeghi H. Mori G. (2020). “Adapting grad-cam for embedding networks,” in IEEE Winter Conference on Applications of Computer Vision (Snowmass, CO: IEEE), 2794–2803.

[B16] Chen Y. Lee W. S. Gan H. Peres N. Fraisse C. Zhang Y.. (2019). Strawberry yield prediction based on a deep neural network using high-resolution aerial orthoimages. Remote Sens. 11, 1584. 10.3390/rs11131584

[B17] Cobb J. N. DeClerck G. Greenberg A. Clark R. McCouch S. (2013). Next-generation phenotyping: requirements and strategies for enhancing our understanding of genotype-phenotype relationships and its relevance to crop improvement. Theor. Appl. Genet. 126, 867–887. 10.1007/s00122-013-2066-0 23471459 PMC3607725

[B18] Cuevas H. E. Zhou C. Tang H. Khadke P. P. Das S. Lin Y.-R.. (2016). The evolution of photoperiod-insensitive flowering in sorghum, a genomic model for panicoid grasses. Mol. Biol. Evol. 33, 2417–2428. 10.1093/molbev/msw120 27335143 PMC4989116

[B19] Deng J. Dong W. Socher R. Li L.-J. Li K. Fei-Fei L. (2009). “Imagenet: a large-scale hierarchical image database,” in Proceedings of the Conference on Computer Vision and Pattern Recognition (Miami, FL), 248–255.

[B20] Dobrescu A. Valerio Giuffrida M. Tsaftaris S. A. (2017). “Leveraging multiple datasets for deep leaf counting,” in IEEE/CVF Conference on Computer Vision and Pattern Recognition Workshops (CVPRW). (Honolulu, HI), 2072–2079.

[B21] Ferentinos K. P. (2018). Deep learning models for plant disease detection and diagnosis. Comput. Electron. Agric. 145, 311–318. 10.1016/j.compag.2018.01.009

[B22] Ge W. Huang W. Dong W. Scott D. Scott M. R. (2018). “Deep metric learning with hierarchical triplet loss,” in Proceedings of the European Conference on Computer Vision (Munich), 269–285.

[B23] Giuffrida M. V. Doerner P. Tsaftaris S. A. (2018). Pheno-deep counter: a unified and versatile deep learning architecture for leaf counting. Plant J. 96, 880–890. 10.1111/tpj.14064 30101442 PMC6282617

[B24] Hadsell R. Chopra S. LeCun Y. (2006). “Dimensionality reduction by learning an invariant mapping,” in Proceedings of the Conference on Computer Vision and Pattern Recognition, Vol. 2 (New York, NY: IEEE), 1735–1742.

[B25] He K. Zhang X. Ren S. Sun J. (2016). “Deep residual learning for image recognition,” in Proceedings of the Conference on Computer Vision and Pattern Recognition (Las Vegas, NV), 770–778.

[B26] Heidary-Sharifabad A. Zarchi M. S. Emadi S. Zarei G. (2021). An efficient deep learning model for cultivar identification of a pistachio tree. Br. Food J. 123, 3592–3609. 10.1108/BFJ-12-2020-110034522735

[B27] Hilley J. L. Weers B. D. Truong S. K. McCormick R. F. Mattison A. J. McKinley B. A.. (2017). Sorghum dw2 encodes a protein kinase regulator of stem internode length. Sci. Rep. 7, 4616. 10.1038/s41598-017-04609-5 28676627 PMC5496852

[B28] Kim W. Goyal B. Chawla K. Lee J. Kwon K. (2018). “Attention-based ensemble for deep metric learning,” in Proceedings of the European Conference on Computer Vision (Munich), 736–751.

[B29] Kingma D. P. Ba J. (2015). “Adam: a method for stochastic optimization,” in International Conference on Learning Representations, eds Y. Bengio and Y. LeCun (San Diego, CA).

[B30] Koirala A. Walsh K. Wang Z. McCarthy C. (2019). Deep learning for real-time fruit detection and orchard fruit load estimation: benchmarking of “mangoyolo”. Precision Agric. 20, 1107–1135. 10.1007/s11119-019-09642-0

[B31] Lang O. Gandelsman Y. Yarom M. Wald Y. Elidan G. Hassidim A.. (2021). Explaining in style: training a gan to explain a classifier in stylespace. arXiv preprint arXiv:2104.13369. 10.1109/ICCV48922.2021.00073

[B32] LeBauer D. Burnette M. A. Demieville J. Fahlgren N. French A. N. Garnett R.. (2020). TERRA-REF, An Open Reference Data Set From High Resolution Genomics, Phenomics, and Imaging Sensors. Available online at: https://datadryad.org/stash/dataset/ 10.5061/dryad.4b8gtht99

[B33] Lim M. G. Chuah J. H. (2018). “Durian types recognition using deep learning techniques,” in 2018 9th IEEE Control and System Graduate Research Colloquium (ICSGRC) (Shah Alam: IEEE), 183–187.

[B34] Liu Y. Wang D. He F. Wang J. Joshi T. Xu D. (2019). Phenotype prediction and genome-wide association study using deep convolutional neural network of soybean. Front. Genet. 10, 1091. 10.3389/fgene.2019.01091 31824557 PMC6883005

[B35] Maimaitijiang M. Sagan V. Sidike P. Hartling S. Esposito F. Fritschi F. B. (2020). Soybean yield prediction from uav using multimodal data fusion and deep learning. Remote Sens. Environ. 237, 111599. 10.1016/j.rse.2019.111599

[B36] Miao C. Guo A. Thompson A. M. Yang J. Ge Y. Schnable J. C. (2021). Automation of leaf counting in maize and sorghum using deep learning. Plant Phenome J. 4, e20022. 10.1002/ppj2.20022

[B37] Mohanty S. P. Hughes D. P. Salathé M. (2016). Using deep learning for image-based plant disease detection. Front Plant Sci. 7, 1419. 10.3389/fpls.2016.01419 27713752 PMC5032846

[B38] Movshovitz-Attias Y. Toshev A. Leung T. K. Ioffe S. Singh S. (2017). “No fuss distance metric learning using proxies,” in Proceedings of the International Conference on Computer Vision (Venice).

[B39] Mural R. V. Grzybowski M. Miao C. Damke A. Sapkota S. Boyles R. E.. (2021). Meta-analysis identifies pleiotropic loci controlling phenotypic trade-offs in sorghum. Genetics 218, iyab087. 10.1093/genetics/iyab087 34100945 PMC9335936

[B40] Murphy R. L. Morishige D. T. Brady J. A. Rooney W. L. Yang S. Klein P. E.. (2014). Ghd7 (ma6) represses sorghum flowering in long days: Ghd7 alleles enhance biomass accumulation and grain production. Plant Genome 7, plantgenome2013.11.0040. 10.3835/plantgenome2013.11.0040

[B41] Nevavuori P. Narra N. Lipping T. (2019). Crop yield prediction with deep convolutional neural networks. Comput. Electron. Agric. 163:104859. 10.1016/j.compag.2019.104859

[B42] Osako Y. Yamane H. Lin S.-Y. Chen P.-A. Tao R. (2020). Cultivar discrimination of litchi fruit images using deep learning. Sci. Hortic. 269:109360. 10.1016/j.scienta.2020.109360

[B43] Ren C. Dulay J. Rolwes G. Pauli D. Shakoor N. Stylianou A. (2021). “Multi-resolution outlier pooling for sorghum classification,” in Agriculture-Vision Workshop in IEEE/CVF Conference on Computer Vision and Pattern Recognition Workshops (CVPRW) (Nashville, TN).

[B44] Sa I. Ge Z. Dayoub F. Upcroft B. Perez T. McCool C. (2016). Deepfruits: a fruit detection system using deep neural networks. Sensors 16, 1222. 10.3390/s16081222 27527168 PMC5017387

[B45] Sandhu K. S. Lozada D. N. Zhang Z. Pumphrey M. O. Carter A. H. (2021). Deep learning for predicting complex traits in spring wheat breeding program. Front. Plant Sci. 11, 2084. 10.3389/fpls.2020.613325 33469463 PMC7813801

[B46] Schroff F. Kalenichenko D. Philbin J. (2015). “Facenet: a unified embedding for face recognition and clustering,” in Proceedings of the Conference on Computer Vision and Pattern Recognition (Boston, MA).

[B47] Schweitzer J. A. Bailey J. K. Fischer D. G. LeRoy C. J. Lonsdorf E. V. Whitham T. G.. (2008). Plant-soil-microorganism interactions: heritable relationship between plant genotype and associated soil microorganisms. Ecology 89, 773–781. 10.1890/07-0337.1 18459340

[B48] Selvaraju R. R. Cogswell M. Das A. Vedantam R. Parikh D. Batra D. (2017). “Grad-cam: visual explanations from deep networks via gradient-based localization,” in Proceedings of the International Conference on Computer Vision (Venice), 618–626.

[B49] Sohn K. (2016). “Improved deep metric learning with multi-class n-pair loss objective,” in Advances in Neural Information Processing Systems (Barcelona), 1857–1865.

[B50] Song H. O. Xiang Y. Jegelka S. Savarese S. (2016). “Deep metric learning via lifted structured feature embedding,” in Proceedings of the Conference on Computer Vision and Pattern Recognition. (Las Vegas, NV).

[B51] Stylianou A. Souvenir R. Pless R. (2019). “Visualizing deep similarity networks,” in IEEE Winter Conference on Applications of Computer Vision (WACV) (Waikoloa, HI: IEEE), 2029–2037.

[B52] Sutskever I. Martens J. Dahl G. Hinton G. (2013). “On the importance of initialization and momentum in deep learning,” in International Conference on Machine Learning (Atlanta, GA: PMLR), 1139–1147.

[B53] Too E. C. Yujian L. Njuki S. Yingchun L. (2019). A comparative study of fine-tuning deep learning models for plant disease identification. Comput. Electron. Agric. 161, 272–279. 10.1016/j.compag.2018.03.032

[B54] Tross M. C. Gaillard M. Zwiener M. Miao C. Grove R. J. Li B.. (2021). 3d reconstruction identifies loci linked to variation in angle of individual sorghum leaves. PeerJ. 9, e12628. 10.7717/peerj.12628 35036135 PMC8710048

[B55] Ubbens J. Cieslak M. Prusinkiewicz P. Parkin I. Ebersbach J. Stavness I. (2020). Latent space phenotyping: automatic image-based phenotyping for treatment studies. Plant Phenomics 2020, 5801869. 10.34133/2020/5801869 33313558 PMC7706325

[B56] Ubbens J. Cieslak M. Prusinkiewicz P. Stavness I. (2018). The use of plant models in deep learning: an application to leaf counting in rosette plants. Plant Methods 14, 1–10. 10.1186/s13007-018-0273-z 29375647 PMC5773030

[B57] Uttam A. Madgula P. Rao Y. Tonapi V. Madhusudhana R. (2017). Molecular mapping and candidate gene analysis of a new epicuticular wax locus in sorghum (sorghum bicolor l. moench). Theor. Appl. Genet. 130, 2109–2125. 10.1007/s00122-017-2945-x 28702690

[B58] Van Horn G. Mac Aodha O. Song Y. Cui Y. Sun C. Shepard A.. (2018). “The inaturalist species classification and detection dataset,” in Proceedings of the Conference on Computer Vision and Pattern Recognition. (Salt Lake City, UT), 8769–8778.

[B59] Vo N. Hays J. (2019). “Generalization in metric learning: Should the embedding layer be embedding layer?” in IEEE Winter Conference on Applications of Computer Vision (WACV) (Waikoloa, HI: IEEE), 589–598.

[B60] Wan S. Goudos S. (2020). Faster r-cnn for multi-class fruit detection using a robotic vision system. Comput. Netw. 168, 107036. 10.1016/j.comnet.2019.107036

[B61] Wang A. X. Tran C. Desai N. Lobell D. Ermon S. (2018). “Deep transfer learning for crop yield prediction with remote sensing data,” in Proceedings of the 1st ACM SIGCAS Conference on Computing and Sustainable Societies. (San Jose, CA), 1–5.

[B62] Wang G. Sun Y. Wang J. (2017). Automatic image-based plant disease severity estimation using deep learning. Comput. Intell. Neurosci. 2017, 2917536. 10.1155/2017/2917536 28757863 PMC5516765

[B63] Wang H. Cimen E. Singh N. Buckler E. (2020). Deep learning for plant genomics and crop improvement. Curr. Opin. Plant Biol. 54, 34–41. 10.1016/j.pbi.2019.12.010 31986354

[B64] Wang Y. Tan L. Fu Y. Zhu Z. Liu F. Sun C.. (2015). Molecular evolution of the sorghum maturity gene ma3. PLoS ON. 10, e0124435. 10.1371/journal.pone.0124435 25961888 PMC4427326

[B65] Wu Y. Li X. Xiang W. Zhu C. Lin Z. Wu Y.. (2012). Presence of tannins in sorghum grains is conditioned by different natural alleles of tannin1. Proc. Natl. Acad. Sci. U.S.A. 109, 10281–10286. 10.1073/pnas.1201700109 22699509 PMC3387071

[B66] Xia J. Zhao Y. Burks P. Pauly M. Brown P. J. (2018). A sorghum nac gene is associated with variation in biomass properties and yield potential. Plant Direct 2, e00070. 10.1002/pld3.70 31245734 PMC6508854

[B67] Xuan H. Souvenir R. Pless R. (2018). “Deep randomized ensembles for metric learning,” in Proceedings of the European Conference on Computer Vision (Munich).

[B68] Yamaguchi M. Fujimoto H. Hirano K. Araki-Nakamura S. Ohmae-Shinohara K. Fujii A.. (2016). Sorghum dw1, an agronomically important gene for lodging resistance, encodes a novel protein involved in cell proliferation. Sci. Rep. 6, 28366. 10.1038/srep28366 27329702 PMC4916599

[B69] Zeiler M. D. Fergus R. (2014). “Visualizing and understanding convolutional networks,” in Proceedings of the European Conference on Computer Vision (Zurich: Springer), 818–833.

[B70] Zhang R. Tian Y. Zhang J. Dai S. Hou X. Wang J.. (2021). Metric learning for image-based flower cultivars identification. Plant Methods 17, 1–14. 10.1186/s13007-021-00767-w 34158091 PMC8220695

[B71] Zhao W. Rao Y. Wang Z. Lu J. Zhou J. (2021). “Towards interpretable deep metric learning with structural matching,” in Proceedings of the International Conference on Computer Vision. (Montreal), 9887–9896.

[B72] Zhou B. Khosla A.A L Oliva A. Torralba A. (2016). “Learning deep features for discriminative localization,” in Proceedings of the Conference on Computer Vision and Pattern Recognition (Las Vegas, NV).

[B73] Zhou B. Khosla A. Lapedriza A. Oliva A. Torralba A. (2015). “Object detectors emerge in deep scene CNNS,” in International Conference on Learning Representations (San Diego, CA).

